# Structural Characterization of a Rhamnogalacturonan I Domain from Ginseng and Its Inhibitory Effect on Galectin-3

**DOI:** 10.3390/molecules22061016

**Published:** 2017-06-18

**Authors:** Huimin Shi, Li Yu, Yun Shi, Jiaojiao Lu, He Teng, Yifa Zhou, Lin Sun

**Affiliations:** Jilin Province Key Laboratory on Chemistry and Biology of Natural Drugs in Changbai Mountain, School of Life Sciences, Northeast Normal University, Changchun 130024, China; shihm356@nenu.edu.cn (H.S.); ylnenu@163.com (L.Y.); shiy028@nenu.edu.cn (Y.S.); lujj112@nenu.edu.cn (J.L.); tengh021@nenu.edu.cn (H.T.); zhouyf383@nenu.edu.cn (Y.Z.)

**Keywords:** pectin, rhamnogalacturonan I, ginseng, galectin-3

## Abstract

A rhamnogalacturonan I domain, named RG-I-3A, was prepared from ginseng pectin by pectinase digestion and chromatography separation. Monosaccharide composition analysis revealed that it was mainly composed of galacturonic acid, rhamnose, galactose, and arabinose in a molar ratio of 32.5:11.2:31.9:16.5, with a molecular weight of 50 kDa. Partial acid hydrolysis, monoclonal antibody detection, and NMR spectra analysis suggested RG-I-3A was composed of →4)-α-Gal*p*A-(1→2)-α-Rha*p*-(1→disaccharide repeating units as backbone, with β-1,4-galactan, α-1,5-arabinan, AG-I, and AG-II side chains substituted via the O-4 of Rha*p*. Galectin-3-mediated hemagglutination and biolayer interferometry assay indicated that RG-I-3A had inhibitory activity on galectin-3. These findings suggest the potential use of this ginseng RG-I domain as a galectin-3 inhibitor in drug development applications.

## 1. Introduction

Pectins are a group of structurally complex polysaccharides that are present in plant cell walls, including homogalacturonan (HG), rhamnogalacturonan I (RG-I), rhamnogalacturonan II (RG-II), xylogalacturonan (XGA), and apiogalacturonan (AGA) [1,2,3]. Compared to other types of pectin, RG-I appears to be highly variable in different plants, and even in different tissues of the same plant [4]. RG-I is usually referred to as a family of structurally diverse polysaccharides composed of a main chain with repeating disaccharide units of (→2)-α-L-Rhap-(1→4)-α-d-GalpA-(1→), with single to polymeric neutral side chains of different types, such as arabinans, galactans, and arabinogalactans-I/II (AG-I/II) [5]. It has been reported that RG-I pectins from different plants have many pharmaceutical activities such as anti-tumor, anti-ulcer, complement-fixing, and immunomodulation activity [6].

Ginseng has been used as a tonic and functional food in the prevention of various diseases in Asia for thousands of years [7]. Polysaccharides are one of the active components of ginseng. Ginseng pectin, accounting for 20% by mass of water-soluble ginseng polysaccharides, mainly contains HG, RG-I, and AG [8]. In recent years, there has been considerable interest in studying the fine structure and the structure-activity relationship of ginseng RG-I pectin. Our research group has isolated a serial of ginseng RG-I fractions by different methods [8,9,10]. These fractions have diverse biological activities including inhibition of cell migration, cancer cell antiproliferative effects, anti-fatigue activity, antidepressant-like effects, and immunomodulatory effects [9,11,12,13,14].

Galectin-3, a β-galactoside-binding lectin, is widely expressed in mammalian cells and involved in various biological processes, such as cell migration, proliferation, and apoptosis [15]. Galectin-3 contributes to tumor progression and metastasisis, and has become a molecular target in the development of anti-cancer therapeutics [16]. In previous work, we has isolated five RG-I domains from ginseng (RG-I-1, RG-I-2, RG-I-3A, RG-I-3B, and RG-I-4) by pectinase digestion and a combination of anion-exchange and size-exclusion chromatography separation [10]. The inhibitory effects of RG-I-2, RG-I-3B, and RG-I-4 against galectin-3 have been studied. Among these fractions, RG-I-4 exhibited the strongest inhibitory effect against galectin-3 and its structure-activity relationship was discussed in a previous paper [17]. As RG-I-3A had monosaccharide composition and molecular weight similar to RG-I-4, therefore, we will focus the present study on analyzing the chemical structure of RG-I-3A by partial acid hydrolysis, monoclonal antibody detection, and NMR spectra, and on assessing its inhibitory effect on galectin-3 by hemagglutination and biolayer interferometry assay. The results presented in this study provide useful information for the further exploration of new potent inhibitor of galectin-3 from ginseng.

## 2. Results and Discussion

### 2.1. Preparation of RG-I-3A from Ginseng Polysaccharides

**Figure 1:**
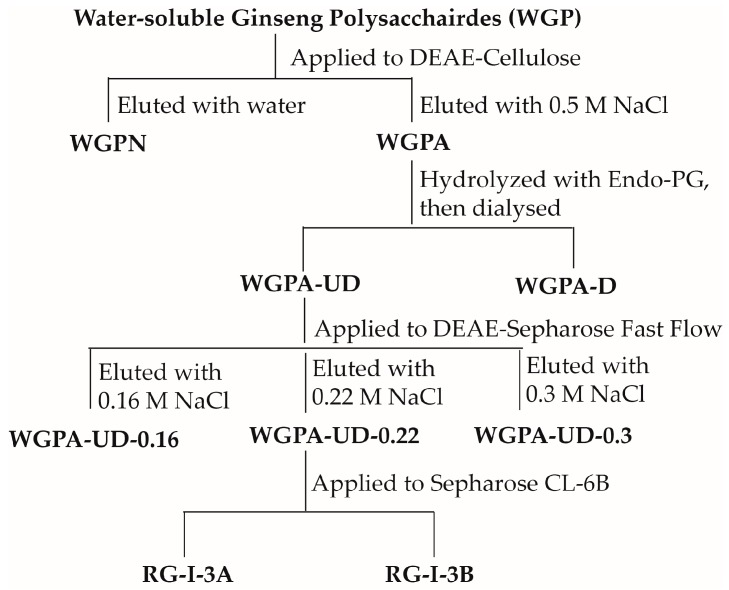
Fractionation and purification protocol of RG-I-3A from ginseng polysaccharides.

Ginseng RG-I-type pectin, RG-I-3A, was prepared according to our published protocol with a minor modification (Figure 1). The yield of RG-I-3A in relation to ginseng pectin (WGPA) was 1.2%, and was 0.25% to water-soluble ginseng polysaccharides (WGP). It was mainly composed of GalA (32.5%), Rha (11.2%), Gal (31.9%), and Ara (16.5%), with traces of GlcA, Man, Glc, and Fuc. The weight average molecular weight of RG-I-3A was 50 kDa, which was consistent with previous results [10].

### 2.2. Partial Acid Hydrolysis of RG-I-3A

Different glycosidic linkages have different susceptibilities to acid hydrolysis. Generally, the linkages between neutral sugars are most susceptible to acid hydrolysis, particularly for furanose, and the linkages between two GalA residues are usually more stable than those of aldobiuronate (GalA-Rha) or pseudo-aldobiuronate (Rha-GalA) [18]. Hence, controlled acid hydrolysis is frequently used to remove side chains in pectin. Here, RG-I-3A was hydrolyzed with 0.1 M trifluoroacetic acid (TFA) for 0.5 to 16 h, and a series of hydrolysis products were obtained after dialysis. The monosaccharide compositions and molecular weights of these products were listed in Table 1.

Compared with RG-I-3A, the Ara and Gal contents decreased rapidly during the first 0.5 h of acid hydrolysis. After 2 h, the Ara residues had disappeared, and the Gal content was approximately half of that in RG-I-3A. Upon prolonged hydrolysis, the content of Gal residues decreased gradually. Till 16 h, only 5.7% of Gal residues were left. In contrast, the contents of GalA and Rha residues increased slowly with the prolongation of hydrolysis. By 16 h, the sample contained mostly GalA and Rha residues in an almost equivalent ratio, which was typical for the backbone of RG-I-type pectin. These results indicated that Ara residues were the most sensitive to acid hydrolysis, then Gal residues, while the linkages between GalA and Rha residues were less sensitive than Ara and Gal residues. Therefore, neutral side chains containing Ara and Gal residues were cleaved along acid hydrolysis, and the backbone of RG-I was obtained. The molecular weight of the remaining RG-I backbone in RG-I-3A-16 was 6.0 kDa, which was 8.3-fold smaller than the parent RG-I-3A, suggesting that RG-I-3A was highly degraded.

### 2.3. Monoclonal Antibody Detection of RG-I-3A Hydrolysis Products

**Figure 2:**
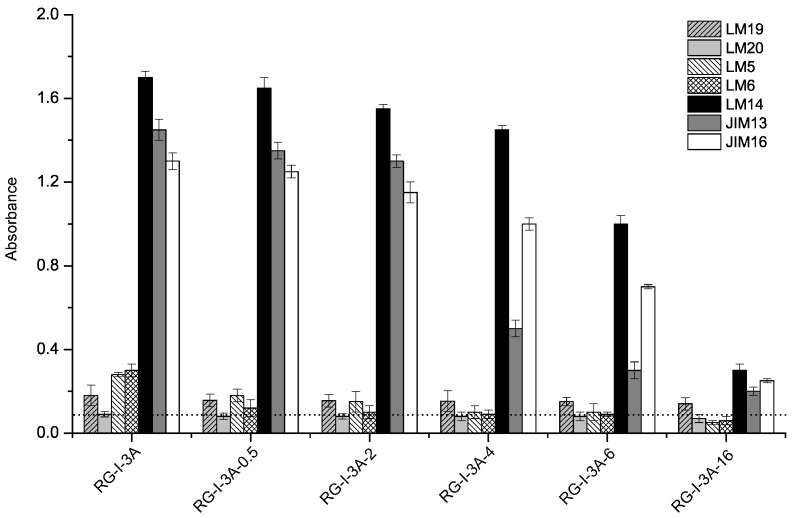
Changes in the binding of monoclonal antibodies to the RG-I-3A-related fractions. The values indicate the means of triplicate experiments. Horizontal dotted lines indicate background signal was lower than 0.1.

During acid hydrolysis, structural changes in RG-I-3A were detected using pectin-directed monoclonal antibodies (Table 2). LM19 is a HG-related monoclonal antibody that could recognize more than four successive de-esterified GalA residues, while LM20 recognizes methyl-esterified HG domains [19]. As shown in Figure 2, RG-I-3A exhibited very weak binding to LM19 and no binding to LM20. With the hydrolysis time increased, the binding of LM19 to RG-I-3A-related fractions did not change much, suggesting that RG-I-3A contained a very small quantity of de-esterified HG domains. LM5 and LM6 recognize β-1,4-galactan and α-1,5-arabinan, respectively [20,21]. They both bound to RG-I-3A (Figure 2). After 0.5 h of acid hydrolysis, their binding to RG-I-3A-0.5 decreased a lot. Till 2 h, the extent of LM5 and LM6 binding to RG-I-3A-2 were very low, suggesting that β-1,4-galactan and α-1,5-arabinan were effectively hydrolyzed. Three AGP glycan-directed antibodies, LM14, JIM13, and JIM16, bound abundantly to RG-I-3A, indicating the presence of type II AG [22,23], with β-1,3-Gal/β-1,6-Gal/β-1,3,6-Gal linkage forms. As hydrolysis was prolonged, the binding of these antibodies to the products decreased gradually. These results indicated that the AG-II side chains were also removed by acid hydrolysis, but were less sensitive than the other neutral side chains. The antibody detection results were consistent with the changes in the monosaccharide compositions for all of the hydrolysis products. Overall, the above results showed that RG-I-3A contained arabinan and galactan or AG-I, as well as AG-II side chains.

### 2.4. NMR Spectra Analysis of RG-I-3A Hydrolysis Products

**Figure 3:**
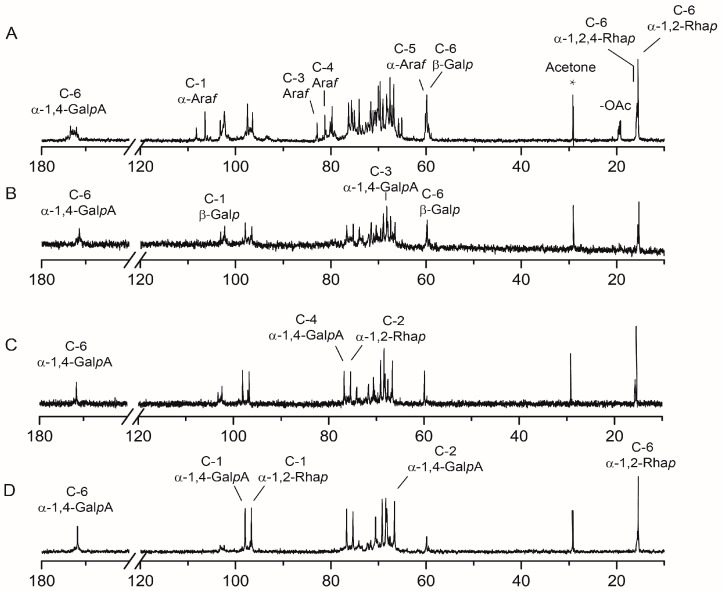
^13^C-NMR spectrum of (**A**) RG-I-3A, (**B**) RG-I-3A-0.5, (**C**) RG-I-3A-2, and (**D**) RG-I-3A-16.

**Figure 4:**
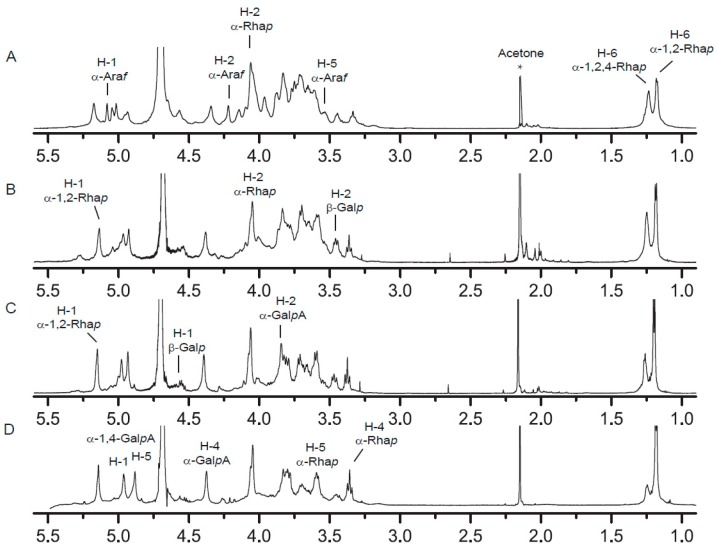
^1^H-NMR spectrum of (**A**) RG-I-3A, (**B**) RG-I-3A-0.5, (**C**) RG-I-3A-2, and (**D**) RG-I-3A-16.

Structural analyses of the acid hydrolyzed products of RG-I-3A were also performed using ^1^H and ^13^C-NMR spectroscopy. Because the quantities of some of the products were not sufficient for NMR studies, we only analyze fractions RG-I-3A-0.5, RG-I-3A-2, and RG-I-3A-16 (Figure 3 and Figure 4). Their chemical shift assignments were listed in Table 3.

In the ^13^C-NMR spectrum of RG-I-3A (Figure 3A), peaks at 108.26, 106.45, and 106.36 ppm were assigned to C-1 of non-reducing terminal residue of Ara*f*, α-1,5-Ara*f*, and α-1,3,5-Ara*f*, respectively. Peaks between 82.90 ppm and 80.13 ppm were attributed to C-2 to C-4 of α-Ara*f*. The anomeric signals at 5.05 and 5.08 ppm in ^1^H-NMR spectrum (Figure 4A) were assigned to α-1,5/1,3,5-Ara*f* and t-Ara*f*, respectively [24]. Following acid hydrolysis to 2 h, these peaks had completely disappeared (Figure 3C and Figure 4C), suggesting that Ara residues were totally cleaved from RG-I-3A. Gal residues of RG-I-3A gave multiple anomeric resonances in the ^13^C-NMR spectrum at 103.22, 102.59, 102.37, and 102.26 ppm, which were attributed to C-1 of β-1,3/1,6-Gal*p*, β-1,4-Gal*p*, β-1,3,6-Gal*p*, and t-β-Gal*p*, respectively. The 75.47 ppm peak was assigned to C-4 of β-1,4-Gal*p*. The 79.36 ppm peak was attributed to C-3 of β-1,3-Gal*p* and β-1,3,6-Gal*p*. The C-6 signal at 67.17 ppm was attributed to β-1,6-Gal*p* and β-1,3,6-Gal*p* [25]. In the ^1^H-NMR spectrum, β-Gal*p* residues showed anomeric signals at 4.54~4.57 ppm. As the hydrolysis time increased, peaks for β-Gal*p* decreased in intensity, and the signals for β-1,4-Gal*p* disappeared faster, indicating that β-1,4-Gal*p* residues were more labile to acid hydrolysis than the other Gal residues. By 16 h, the β-Gal*p* resonances in RG-I-4-16 had almost completely disappeared (Figure 3D and Figure 4D). These results confirmed that the Ara residue was the most sensitive to acid hydrolysis, then Gal residue, which was consistent with the monosaccharide composition and antibody detection analyses. NMR spectroscopy further indicated that the Ara and Gal residues in RG-I-3A existed as arabinan and galactan or AG-I, as well as AG-II side chains.

RG-I-3A showed a broad peak at around 172.44 ppm in the ^13^C-NMR spectrum, which was attributed to the α-1,4-Gal*p*A C-6 groups. C-1 and H-1 resonance signals of α-1,4-Gal*p*A appeared at 97.51 and 5.02 ppm, respectively. Methyl signals from the α-1,4-Gal*p*A acetyl groups were clearly evident at 22.0 ppm in the ^13^C-NMR spectrum [26], suggesting that RG-I-3A was highly acetylated. However, this peak had disappeared after 0.5 h of acid hydrolysis (Figure 3B), indicating that the acetyl groups were removed by weak acid hydrolysis. No methyl group of the carboxylic acid methyl ester was observed in RG-I-3A as the absence of signals between 50 and 55 ppm in the ^13^C-NMR spectrum. The anomeric carbon and proton signals of α-Rha*p* were clearly identified at 96.44 ppm and 5.17 ppm in RG-I-3A. The C-6 groups of α-1,2-Rha*p* and α-1,2,4-Rha*p* gave signals at 15.50 ppm and 15.72 ppm, and their H-6 groups showed signals at 1.18 and 1.24 ppm, respectively [27]. Both integrated C-6 and H-6 peak intensity ratio of α-1,2,4-Rha*p*/(α-1,2-Rha*p*+α-1,2,4-Rha*p*) decreased gradually from 0.5 h to 16 h hydrolysis. For RG-I-3A-16 (Figure 3D and Figure 4D), the 15.72 ppm and 1.24 ppm signals for C-6 and H-6 of α-1,2,4-Rha*p* were barely apparent. These spectral changes suggested that the substitution at Rha C-4 decreased progressively as the time of hydrolysis increased and was almost completely removed by 16 h, leaving only the RG-I backbone.

### 2.5. Inhibitory Effect of RG-I-3A on Galectin-3

Galectin-3 is a β-galactoside binding lectin associated with various cell processes. The binding between galectin-3 and pectin make it a potential galectin-3 inhibitor with applications in preventing cancer, carcinogenesis, and many other diseases. In this paper, the inhibitory effect of RG-I-3A on galectin-3 was assessed by a hemagglutination and biolayer interferometry assay.

**Figure 5:**
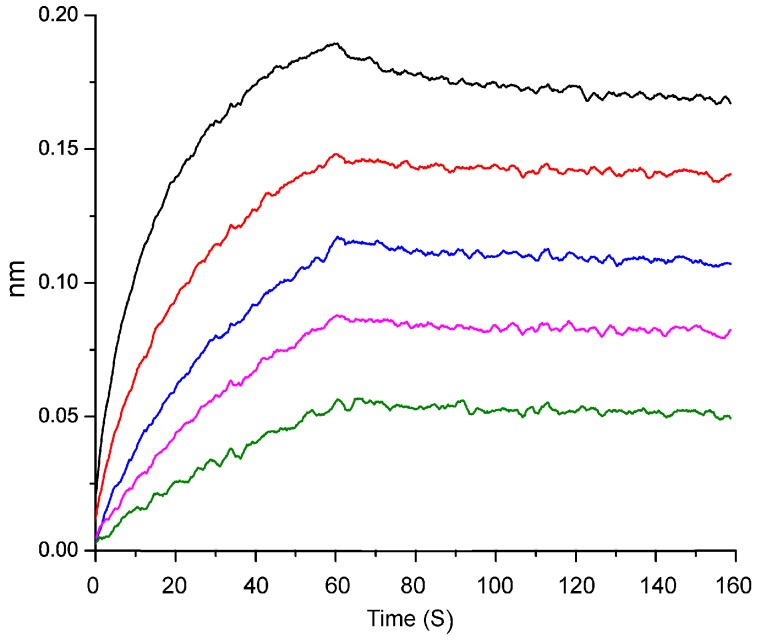
Biolayer interferometry analysis of the binding affinity of RG-I-3A to galectin-3. Association and dissociation curves are presented, and KD values were analyzed using the Fortebio Data Analysis Software 7.0. RG-I-3A concentrations (from top to bottom): 1.6, 0.8, 0.4, 0.2, 0.1 μM.

The galectin-3-mediated hemagglutination assay is widely used to evaluate inhibitory effects of galectin-3 ligands by measuring the minimum inhibitory concentration (MIC) [28]. RG-I-3A exhibited a very strong inhibitory effect on galectin-3 with a MIC value of 0.6 ± 0.06 μg/mL. The inhibitory activity was similar to that of modified citrus pectin (MCP, MIC 0.6 ± 0.05 μg/mL) and stronger than that of potato galactan (MIC 9.0 ± 1.1 μg/mL), which are two well established galectin-3 ligands [17]. Compared with other RG-I domains from ginseng, the inhibitory activity of RG-I-3A on galectin-3 was stronger than RG-I-2 (MIC 60 ± 3.8 μg/mL) and RG-I-3B (120 ± 6.3 μg/mL), but a little weaker than RG-I-4 (MIC 0.25 ± 0.02 μg/mL) presented in our previous work [17]. The binding kinetics of RG-I-3A with galectin-3 was further determined by biolayer interferometry using an Ni-NTA sensor. The association and dissociation curves of different RG-I-3A dilutions were shown in Figure 5. Non-specific binding has been eliminated because, in the absence of galectin-3, minimal pectin bound even at the highest concentrations used in the study. The dissociation constant (*K_D_*) was calculated to be 28 nM, indicating a strong binding affinity for RG-I-3A to galectin-3, consistent with the result of the galectin-3-mediated hemagglutination assay. The binding affinity for RG-I-3A to galectin-3 was higher than that of MCP (*K_D_* 1151 nM) and potato galactan (*K_D_* 79 nm), but lower than RG-I-4 (*K_D_* 13 nM) [29]. These data suggested a potential use of RG-I-3A from ginseng as a new galectin-3 inhibitor.

In our previous study, we have found RG-I-4 was a potent galectin-3 inhibitor which showed a very strong inhibition effect on galectin-3 [17]. On the way, a new inhibitor of galectin-3, RG-I-3A, was found to have a little weaker inhibition effect than RG-I-4 on galectin-3, but much stronger than RG-I-2 and RG-I-3B. The activity differences among these ginseng RG-I domains should be related to their sugar compositions, molecular weight, and structure differences. The order of Gal content is RG-I-3A (31.9%) > RG-I-4 (19.5%) > RG-I-2 (12.4%) ~ RG-I-3B (13.7%) [10]. High Gal content usually caused high inhibitory activity of pectin on galectin-3 [17]. The inhibitory activity was not consistent with the Gal content in RG-I-3A and RG-I-4. This might be caused by Gal/Ara ratio in RG-I-3A (1.93), lower than in RG-I-4 (2.12). The lower Gal/Ara ratio in RG-I-3A reflected that more Ara residues might be connected to the end of the side chain. Thus, Ara prohibited Gal interaction with galectin-3. Besides, the molecular weight might be an important factor to affect the activity. The molecular weight of RG-I-3A (50 kDa) and RG-I-4 (60 kDa) were higher than those in RG-I-2 (4 kDa) and RG-I-3B (6 kDa) [10], which resulted in a different inhibitory activity on galectin-3.

## 3. Materials and Methods

### 3.1. Materials

Anti-rat IgG and horseradish peroxidase (HRP) were purchased from Sigma-Aldrich (St. Louis, MO, USA). The series of monoclonal antibodies used to assess the polymers present in the isolated RG-I fractions were kindly provided by Professor Paul Knox from the University of Leeds. Sephadex G-25, DEAE-Sepharose Fast Flow, and Sepharose CL-6B were purchased from GE Healthcare (Uppsala, Sweden). All other reagents and chemicals were commercially available, of analytical grade, and produced in China.

### 3.2. Preparation of RG-I-3A from Ginseng Polysaccharides

RG-I-3A was prepared from water-soluble ginseng polysaccharides (WGP) as previously described with little modification [10]. Briefly, WGP was applied to a DEAE-Cellulose column and eluted with water to give a neutral fraction (WGPN) and with 0.5 M NaCl to give an acidic fraction (WGPA). The WGPA fraction was hydrolyzed using endo-polygalacturonase (Sigma-Aldrich, St. Louis, MO, USA), and the hydrolysates were dialyzed (MWCO 3500 Da) to remove oligosaccharides. The polymeric fraction inside of the dialysis tubes were separated by DEAE-Sepharose Fast Flow column, eluted with 0.16 M, 0.22 M, and 0.3 M NaCl, respectively. The fraction eluted by 0.22 M NaCl was further purified on Sepharose CL-6B column, giving two fractions named RG-I-3A and RG-I-3B.

### 3.3. Molecular Weight Distribution and Monosaccharide Composition Analysis

The homogeneity and molecular weights were estimated using a HPGPC-linked gel permeation column of TSK-gel G-3000 PWXL (7.8 × 300 mm, TOSOH, Japan) and eluted with 0.2 M NaCl at a flow rate of 0.6 mL/min at 35.0 ± 0.1 °C. The monosaccharide compositions were analyzed by HPLC, as previously described. Briefly, the sample (2 mg) was first hydrolyzed using anhydrous methanol containing 2 M HCl at 80 °C for 16 h and then with 2 M TFA at 120 °C for 1 h. The released monosaccharides were derivatized to 1-phenyl-3-methyl-5-pyrazolone (PMP) derivatives and subsequently analyzed by HPLC on a Shim-pak VP-ODS column (150 × 4.6 mm i.d.) with a guard column on a Shimadzu HPLC [8].

### 3.4. Partial Acid Hydrolysis

RG-I-3A (30 mg) were hydrolyzed by 0.1 M TFA (1.5 mL) at 80 °C for 0.5, 2, 4, 6, and 16 h, respectively, and 1 mL of absolute ethanol was added immediately to evaporate the TFA and incubated at 40 °C in a water bath. The hydrolysates were dialyzed (MWCO = 1,000 Da) with distilled water for 24 h and then lyophilized. A series of hydrolyzed products were obtained, namely RG-I-3A-0.5, RG-I-3A-2, RG-I-3A-4, RG-I-3A-6, and RG-I-3A-16, respectively, with the suffix number indicating the period of hydrolysis.

### 3.5. ELISA Assay

The ELISA assay was performed as previously described [30]. The series of acid hydrolysis products of RG-I-3A (50 μg/mL) in phosphate-buffered saline (PBS) were coated on 96-well Nunc-Immuno MaxiSorp microtiter plates overnight at 4 °C. The coating solutions were removed and 200 μL/well of 3% (*w*/*v*) milk protein in PBS (MP/PBS) were added to block the plates for 2 h at room temperature to prevent non-specific binding. The plates were washed, and then 100 μL/well of the primary antibody were then added at a 1:5 dilution in MP/PBS. Following incubation for 1.5 h, the plates were washed, and the wells were incubated with anti-rat IgG coupled to horseradish peroxidase (HRP) for an additional 1.5 h. After washing with PBS, 100 μL/well of freshly prepared HRP-substrate (18 mL of de-ionized water, 2 mL of 1 M sodium acetate buffer, pH 6.0, 200 μL of tetramethylbenzidine, and 20 μL of 6% (*v*/*v*) hydrogen peroxide) were added. The reaction was stopped after 5 min by the addition of 30 μL/well of 2 N H_2_SO_4_. Antibody binding was determined by measuring the absorbance at 450 nm in a micro-plate reader.

### 3.6. NMR Spectra Analysis

^13^C and ^1^H-NMR spectra were recorded using a Bruker 5 mm broadband observe probe at 20 °C with a Bruker Avance 600 MHz spectrometer (Bruker, Rheinstetten, Germany), operating at 600 MHz for ^1^H-NMR and 150 MHz for ^13^C-NMR. The sample (20 mg) was dissolved in D_2_O (99.8%, 0.5 mL), freeze-dried, re-dissolved in D_2_O (0.5 mL), and centrifuged to remove the excess sample. All the data were analyzed using standard Bruker software. Chemical shifts were given in ppm, with acetone as an internal chemical shift reference [8].

### 3.7. Galectin-3-Mediated Hemagglutination Assay

This assay was performed as previously described [17]. Briefly, each well of a microtiter V plate contained 25 μL of 1% bovine serum albumin (BSA), 25 μL of 0.15 M NaCl (control) or the test sample in the same solution, 25 μL of 12.5 μg/mL galectin-3, and 25 μL of a 4% (*v*/*v*) chicken erythrocyte suspension. All dilutions were done in PBS. Agglutination was allowed to proceed for 90 min at 4 °C. The MIC of the test sample was determined by a serial of dilution. The result was the average of three independent experiments.

### 3.8. Biolayer Interferometry Assay

The affinity of RG-I-3A for galectin-3 was measured by using a ForteBio Octet RED 96 instrument (ForteBio, Fremont, CA, USA). Ni-NTA biosensor (ForteBio, Fremont, CA, USA) was hydrated with PBS for 10 min prior to performing the experiment. The concentration of His-tagged galectin-3 was 10 μg/mL, and monitoring was as follows: initial baseline for 60 s, loading for 100 s, baseline for 60 s, association for 60 s, and dissociation for 100 s. The regeneration buffer was 10 mM glycine (pH 2.0), and re-charging (10 mM NiCl_2_ in H_2_O) was done for 60 s. The kinetics buffer was PBS (pH 7.2). To determine binding kinetics, five concentrations of RG-I-3A were dissolved in PBS (0.1, 0.2, 0.4, 0.8, 1.6 μM). Data were analyzed using ForteBio Data Analysis Software 7.0 [29].

## 4. Conclusions

In this study, a RG-I domain (RG-I-3A) was prepared from ginseng, and its detailed chemical structure were analyzed by partial acid hydrolysis, monoclonal antibody detection, and NMR spectra analysis. RG-I-3A was composed of →4)-α-Gal*p*A-(1→2)-α-Rha*p*-(1→disaccharide repeating units as backbone, with branches of β-1,4-galactan, α-1,5-arabinan, and AG-I and AG-II side chains substituted on the backbone via the O-4 of Rha*p*. A galectin-3-mediated hemagglutination assay and biolayer interferometry assay indicated that RG-I-3A had a strong inhibitory effect on galectin-3. This finding suggests that RG-I-3A might be exploited as a new potent galectin-3 inhibitor from ginseng.

## Figures and Tables

**Table 1:** Molecular weights and sugar compositions of RG-I-3A fractions following acid hydrolysis.

Fraction	Mw (kDa)	Sugar Compositions (mol %)						
		GalA	Rha	Gal	Ara	GlcA	Glc	Man
RG-I-3A	50.0 ± 1.05	32.5±0.76	11.2 ± 0.38	31.9 ± 0.66	16.5 ± 0.35	3.0 ± 0.15	1.9 ± 0.30	2.1 ± 0.22
RG-I-3A-0.5	43.0 ± 0.96	33.2 ± 0.55	30.3 ± 0.47	21.6 ± 0.54	8.0 ± 0.30	2.1 ± 0.21	2.6 ± 0.26	1.6 ± 0.25
RG-I-3A-2	21.5 ± 0.88	37.8 ± 0.45	37.2 ± 0.45	20.3 ± 0.50	---	2.4 ± 0.17	1.7 ± 0.19	1.7 ± 0.20
RG-I-3A-4	7.2 ± 0.80	40.2 ± 0.48	39.6 ± 0.51	14.8 ± 0.45	---	2.5 ± 0.17	1.9 ± 0.15	0.9 ± 0.15
RG-I-3A-6	6.3 ± 0.55	41.2 ± 0.52	40.1 ± 0.36	13.3 ± 0.37	---	2.9 ± 0.22	2.2 ± 0.23	0.3 ± 0.09
RG-I-3A-16	6.0 ± 0.60	45.6 ± 0.61	44.8 ± 0.44	5.7 ± 0.32	---	1.9 ± 0.15	---	1.9 ± 0.18

**Table 2:** Cell wall glycan-directed monoclonal antibodies.

Pectin	Antibody	Antigen Epitope	Reference
HG ^a^	LM19	Partially Me-HG/de-esterified HG	[19]
HG ^a^	LM20	Partially Me-HG	[19]
RG-I ^b^	LM5	(1→4)-β-D-galactan (~ four (1→4)-β-Gal)	[20]
RG-I ^b^	LM6	Linear (1→5)-α-L-arabinan (~five (1→5)-α-Ara)	[21]
AGP ^c^	LM14	AGP glycan (AG-II)	[22]
AGP ^c^	JIM13	AGP glycan (AG-II)	[23]
AGP ^c^	JIM16	AGP glycan (AG-II)	[23]

^a^ HG: homogalacturonan; ^b^ RG-I: rhamnogalacturonan I; ^c^ AGP: arabinogalactan-protein.

**Table 3:** ^13^C and ^1^H-NMR spectral assignments of RG-I-3A, RG-I-3A-0.5, RG-I-3A-2, and RG-I-3A-16.

Fraction	Sugar Residues	Chemical Shifts, δ (ppm)					
		C-1/H-1	C-2/H-2	C-3/H-3	C-4/H-4	C-5/H-5	C-6/H-6
RG-I-3A	→4)-α-Gal*p*A-(1→	97.51/5.02	66.78/3.87	68.26/3.83	76.30/4.34	70.05/4.94	172.44/--
	→2)-α-Rha*p*-(1→	96.44/5.17	75.63/4.06	69.05/3.77	69.65/3.33	67.57/3.53	15.50/1.18
	→2,4)-α-Rha*p*-(1→	96.44/5.17	75.63/4.06	69.05/3.75	76.10/3.54	67.57/3.55	15.72/1.24
	t-β-Gal*p*-(1→	102.26/4.54	70.84/3.45	73.39/3.61	67.99/4.09	74.59/3.65	59.85/3.73
	→4)-β-Gal*p*-(1→	102.59/4.54	71.72/3.45	72.73/3.61	75.47/4.14	73.39/3.70	59.85/3.73
	→3)-β-Gal*p*-(1→	103.22/4.57	71.74/3.45	79.36/--	67.99/4.09	74.59/3.65	59.94/3.73
	→6)-β-Gal*p*-(1→	103.22/4.55	71.74/3.45	73.39/3.61	67.99/4.09	74.59/3.66	67.17/4.14
	→3,6)-β-Gal*p*-(1→	102.37/4.57	71.74/3.45	79.36/--	67.99/4.09	74.59/3.66	67.17/4.14
	→5)-α-Ara*f*-(1→	106.45/5.05	81.06/4.22	79.75/--	81.67/4.14	65.83/3.88	--/--
	→3,5)-α-Ara*f*-(1→	106.36/5.05	79.85/4.22	82.90/--	80.13/4.14	65.12/3.88	--/--
	t-α-Ara*f*-(1→	108.26/5.08	81.06/4.22	79.75/--	81.27/4.14	60.13/3.61	--/--
RG-I-3A-0.5	→4)-α-Gal*p*A-(1→	97.96/4.96	66.50/3.84	68.96/3.83	76.66/4.38	70.47/4.93	171.45/--
	→2)-α-Rha*p*-(1→	96.61/5.14	75.30/4.09	69.62/3.79	70.13/3.36	68.24/3.58	15.30/1.18
	→2,4)-α-Rha*p*-(1→	96.86/5.14	75.30/4.09	69.61/3.78	76.05/3.54	68.24/3.59	15.54/1.25
	t-β-Gal*p*-(1→	102.28/4.54	70.25/3.46	73.28/3.65	67.06/4.00	73.35/3.68	59.76/3.78
	→6)-β-Gal*p*-(1→	103.12/4.55	69.86/3.46	73.28/3.65	67.06/4.00	73.35/3.93	67.06/4.14
	→3,6)-β-Gal*p*-(1→	102.54/4.57	69.86/3.47	80.02/--	67.06/4.00	73.35/3.93	67.06/4.14
RG-I-3A-2	→4)-α-Gal*p*A-(1→	98.08/4.93	66.65/3.85	68.38/3.80	76.78/4.38	70.62/4.81	171.61/--
	→2)-α-Rha*p*-(1→	96.74/5.15	75.42/4.06	69.11/3.78	70.62/3.31	68.18/3.59	15.43/1.22
	→2,4)-α-Rha*p*-(1→	96.99/5.15	75.42/4.06	69.11/3.78	76.22/3.55	68.18/3.61	15.68/1.27
	t-β-Gal*p*-(1→	102.41/4.53	70.13/3.53	73.75/3.66	67.54/4.00	73.42/3.68	59.89/3.76
	→6)-β-Gal*p*-(1→	103.25/4.55	69.65/3.53	73.75/3.66	67.54/4.00	73.42/3.94	67.75/4.11
	→3,6)-β-Gal*p*-(1→	102.64/4.56	69.65/3.55	--/--	67.54/4.02	73.42/3.94	67.75/4.11
RG-I-3A-16	→4)-α-Gal*p*A-(1→	98.00/4.96	66.65/3.81	68.31/3.77	76.70/4.38	70.62/4.88	171.86/--
	→2)-α-Rha*p*-(1→	96.69/5.14	75.37/4.06	68.47/3.69	69.24/3.31	68.21/3.59	15.44/1.22
